# Hydrogen overproducing nitrogenases obtained by random mutagenesis and high-throughput screening

**DOI:** 10.1038/srep38291

**Published:** 2016-12-02

**Authors:** Emma Barahona, Emilio Jiménez-Vicente, Luis M. Rubio

**Affiliations:** 1 Centro de Biotecnología y Genómica de Plantas, Universidad Politécnica de Madrid (UPM) - Instituto Nacional de Investigación y Tecnología Agraria y Alimentaria (INIA), Campus Montegancedo UPM, 28223-Pozuelo de Alarcón, Madrid, Spain

## Abstract

When produced biologically, especially by photosynthetic organisms, hydrogen gas (H_2_) is arguably the cleanest fuel available. An important limitation to the discovery or synthesis of better H_2_-producing enzymes is the absence of methods for the high-throughput screening of H_2_ production in biological systems. Here, we re-engineered the natural H_2_ sensing system of *Rhodobacter capsulatus* to direct the emission of LacZ-dependent fluorescence in response to nitrogenase-produced H_2_. A *lacZ* gene was placed under the control of the *hupA* H_2_-inducible promoter in a strain lacking the uptake hydrogenase and the *nifH* nitrogenase gene. This system was then used in combination with fluorescence-activated cell sorting flow cytometry to screen large libraries of nitrogenase Fe protein variants generated by random mutagenesis. Exact correlation between fluorescence emission and H_2_ production levels was found for all automatically selected strains. One of the selected H_2_-overproducing Fe protein variants lacked 40% of the wild-type amino acid sequence, a surprising finding for a protein that is highly conserved in nature. We propose that this method has great potential to improve microbial H_2_ production by allowing powerful approaches such as the directed evolution of nitrogenases and hydrogenases.

Biological H_2_ production is a promising source of renewable energy. Microorganisms produce H_2_ by the activity of nitrogenases and hydrogenases^1^. Nitrogenases catalyze the reduction of N_2_ with the limiting stoichiometry N_2_+8 H^+^ + 8e^−^+16MgATP+16H_2_O↔H_2_+2NH_3_+16MgADP+16P_i_ in a process known as biological nitrogen fixation, which produces H_2_ as a by-product^2^. On the other hand, hydrogenases catalyze the reversible 2 H^+^ +  2e^−^↔H_2_ reaction. H_2_ metabolism has been well studied in purple non-sulfur bacteria (PNS), a group of microorganisms notable for their metabolic versatility. PNS can grow photoautotrophically, photoheterotrophically, chemoorganotrophically, and chemolitotrophically with H_2_ as an electron donor and O_2_ as an electron acceptor^3^. H_2_ production by PNS generally occurs during photoheterotrophic anaerobic growth and is mainly due to nitrogenase^4^.

*Rhodobacter capsulatus*, the model PNS used in this study^5^, carries two genetically distinct nitrogenases (a Mo-nitrogenase and an Fe-only nitrogenase)^6^, which are differentially expressed depending on the Mo availability in the medium^7^, and two hydrogenases, a membrane-bound [Ni-Fe] hydrogenase for H_2_ uptake and a cytosolic [Ni-Fe] hydrogenase for H_2_ sensing^8^. Nitrogenases are two-component metalloproteins comprising an N_2_-reducing dinitrogenase (MoFe protein) and a dinitrogenase reductase (Fe protein) acting as an obligate electron donor^9^. These components are encoded by *nifDK* and *nifH*, respectively, in the case of the Mo-nitrogenases, and by *anfDGK* and *anfH* in the case of the Fe-only nitrogenases^10^. The uptake hydrogenase is a heterodimer of the *hupA* and *hupB* (formerly *hupS* and *hupL*) gene products^11^, in which activity is linked to the respiratory chain by the cytochrome *b*-containing protein HupC. Structural, maturation-related and regulatory genes for the uptake hydrogenase are clustered in the genome of *R. capsulatus*^12^ (Fig. S1).

In *R. capsulatus*, a two-component signal transduction system activates the transcription of the *hup* gene cluster in the presence of H_2_ 13. The H_2_-sensing system comprises three elements: a cytosolic [Ni-Fe] hydrogenase, HupUV; a histidine kinase, HupT; and a response regulator, HupR. In the absence of H_2_, HupUV and HupT interact, causing HupT autophosphorylation and the transfer of a phosphate group to HupR, which in this state is unable to activate the transcription. In the presence of H_2_, HupUV binds H_2_ and HupT is released. In this state, phosphotransfer between HupT and HupR is not favored, and the unphosphorylated HupR binds to promoter DNA and activates the transcription of uptake hydrogenase genes. Similar regulatory systems are found in other bacterial species such as *Bradyrhizobium japonicum*^14^ and *Ralstonia eutropha*^15^.

Due to its great commercial significance, nitrogenase has been the subject of extensive biochemical, genetic and structural analyses. Nonetheless, it has proven difficult to find or engineer strains of microbes carrying nitrogenases with significantly increased H_2_ production efficiency. In this work, we present a method for the high-throughput selection of nitrogenase variants with enhanced H_2_ production. An *R. capsulatus* strain has been re-engineered to generate a fluorescent signal in response to nitrogenase-produced H_2_. A combination of *nifH* random mutagenesis and fluorescence-activated cell sorting (FACS) is then used to select the H_2_-overproducing nitrogenase variants in the *R. capsulatus* sensor strain (Fig. 1A).

## Results

### Genetic modules for the selection of H_2_-overproducing nitrogenase variants

The construction of two genetic modules was required to perform high-throughput experiments to obtain nitrogenase variants with improved H_2_ production: a module expressing over a million random variants of dinitrogenase reductase (NifH) per experiment and a reporter module directing the emission of a visible signal in response to H_2_.

**Figure 1:**
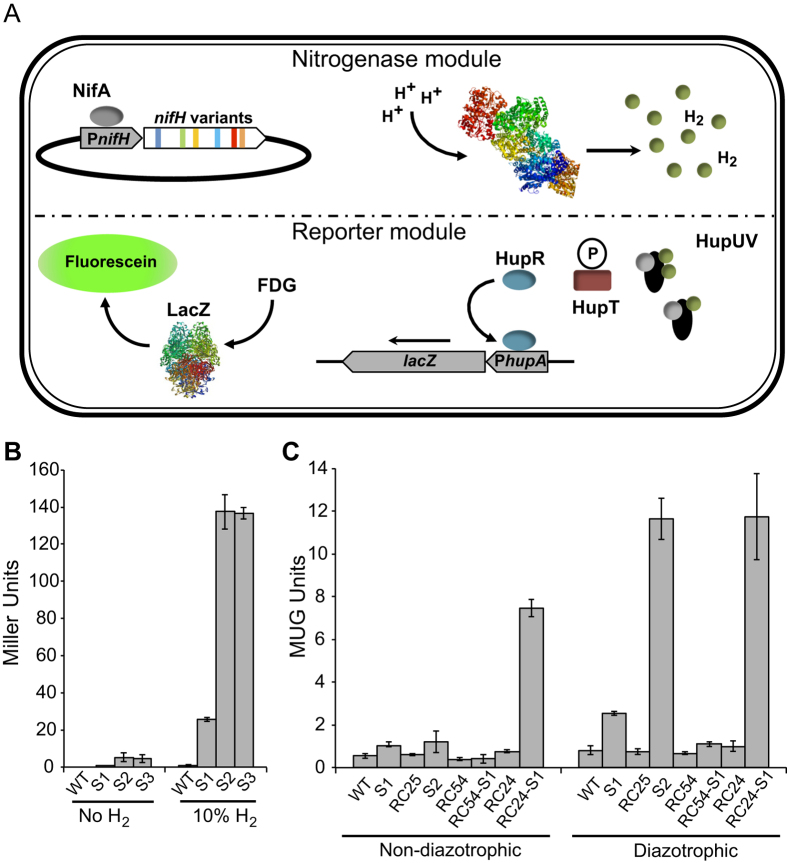
Construction of a biosensor to select H_2_-overproducing nitrogenase variants. (**A**) Biosensor design. NifA-dependent expression libraries of *nifH* variants are randomly generated by error-prone PCR. In each *R. capsulatus* cell, HupUV detects H_2_ produced by a nitrogenase variant, and the signal is transduced to regulate the expression of *lacZ*, which serves as a reporter by catalyzing fluorescein isothiocyanate formation. FACS is then used to sort the cells and select those emitting fluorescence in a range at least an order of magnitude larger than the population average. The selected cells are finally subjected to further measurements of the fluorescence emission and H_2_ production. (**B**) Response of sensor strains to 10% H_2_ added to the culture gas phase. (**C**) Response of sensor strains to H_2_ produced by nitrogenase activity. Data represent the mean ± SD (n = 4). Strains: S1 (P*hupA*::*lacZ*), RC25 (Δ*hupAB*), S2 (P*hupA*::*lacZ*, Δ*hupAB*), RC54 (Δ*hupR*), RC24 (Δ*hupT*), RC54-S1 (P*hupA*::*lacZ*, Δ*hupR*), RC24-S1 (P*hupA*::*lacZ*, Δ*hupT*), and S3 (P*hupA*::*lacZ*, Δ*hupAB*, Δ*nifH*).

The reporter module was constructed in four steps. In a first step, an 874-bp DNA fragment comprising a promoter sequence upstream of *hupA*^16^ was translationally fused to *lacZ* in the replicative vector pMP220 to generate pRHB502. As illustrated in Fig. 1A, the expression from *hupA* promoter is activated by HupR in response to H_2_. The *in vivo* β-galactosidase activity of *R. capsulatus* cells harboring pRHB502 (RC4) was 600-fold higher than that of the control strain RC3 (carrying pMP220) and responded positively to the presence of 10% H_2_ in the culture gas phase, which confirmed the induction of the transcription from P*hupA* by H_2_ (Fig. S2A).

In a second step, the reporter dose was adjusted by integrating P*hupA::lacZ* between *hypF* and *hupA* in the *R. capsulatus* chromosome to generate the S1 strain (Fig. S1). The S1 strain exhibited a much lower β-galactosidase activity background level and a larger fold increase in the activity in response to the external H_2_ than RC4 (Fig. S2B).

In a third step, the reporter response to H_2_ was adjusted by mutating the genes involved in the H_2_ signal transduction pathway and metabolism. S1 derivative strains lacking *hupAB* structural genes for the uptake-hydrogenase (RC25-S1, also termed S2), the H_2_ response regulator encoding gene *hupR* (RC54-S1), or the histidine kinase gene *hupT* (RC24-S1) were generated, and their responses to H_2_ were analyzed (Fig. 1B and Fig. S3), obtaining the following results. First, the response to 10% exogenous H_2_ improved 5-fold in the Δ*hupAB* strain S2 compared to in S1; second, the constitutive activation of P*hupA* was observed in the Δ*hupT* strain RC24-S1; and third, no response to H_2_ was observed in the Δ*hupR* strain RC54-S1. These results were in agreement with previous reports^13^,^16^,^17^,^18^ indicating proper control by the *R. capsulatus* H_2_-sensing system and therefore permitting further H_2_ sensor development through the use of strain S2.

The deletion of *hupAB* genes in the RC25 strain completely eliminated its *in vivo* uptake hydrogenase activity (nil compared to 2504 ± 450 nmol H_2_ h^−1^ OD_600_^−1^ in the wild-type strain). Thus, in contrast to the wild-type strain, the S2 sensor strain (derived from RC25) evolved high levels of H_2_ under diazotrophic growth conditions (Fig. S2C) in spite of having similar levels of *in vivo* nitrogenase activity (Fig. S2D). In addition, the X-gal and H_2_-dependent signal-to-noise ratio was much better in S2 than in S1 cultures (Fig. S2E). It was therefore concluded that the elimination of the uptake hydrogenase activity would facilitate the detection of intracellular H_2_ produced during the nitrogen fixation process.

The S2 response to the H_2_ produced by the nitrogenase was evaluated in intact bacterial cells by using a fluorescent MUG-dependent β-galactosidase activity assay^19^, which permitted high-throughput growth and screening in a 96-well format as well as the recovery of viable selected clones. *R. capsulatus* strains were grown in 96-well microplates both under non-diazotrophic and diazotrophic conditions inside a glovebox, and their β-galactosidase activities were determined. Figure 1C shows that the β-galactosidase activity in S2 was 15-fold higher under diazotrophic conditions compared to non-diazotrophic conditions, validating its use as a biosensor. Importantly, all tested strains with mutations in H_2_ the metabolism or signal transduction pathway responded identically to nitrogenase-produced H_2_ and exogenous added H_2_ (compare Fig. 1C to Fig. S3).

Finally, the removal of endogenous *nifH* was necessary to use the sensor in combination with the genetic module consisting of an expression library of *nifH* variants (Fig. S4). The S3 sensor strain (Δ*nifH* Δ*hupAB* P*hupA::lacZ*) β-galactosidase activity levels in response to H_2_ were identical to those of S2 (Fig. 1B), demonstrating that the absence of *nifH* did not modify the capacity to detect H_2_. Importantly, the introduction of the pRHB576 expression vector carrying a wild-type copy of *nifH* under the control of its own promoter restored the *in vivo* nitrogenase activity of strain S3 (Fig. S5), validating its use to screen the activity of the *nifH* variants in S3. Interestingly, a general decrease in the nitrogenase activities was observed in the sensor S3-derived strains with respect to the wild-type *R. capsulatus*. This effect was associated with carrying the expression vector pBBR1MCS-3 (Fig. S6).

The second genetic module consisted of an expression library of randomly generated nitrogenase variants. The mutation analysis was initially constrained to *nifH* because the limiting steps in the nitrogenase catalysis are the dissociation of the NifH and NifDK components and the release of Pi from NifH^2^. Random *nifH* variants (*nifHv*) having an average of 5 amino acid changes (Table S1) were generated by error-prone PCR, ligated into pRHB602, and introduced into *E. coli* DH5α to obtain P*nifH*::*nifH* expression libraries (~ 4 × 10^6^ clones per library). No obvious mutational hotspots were observed after sequencing 20 *nifH* variants per library.

Finally, the two genetic modules were combined by introducing the *nifH*v-expression libraries into *R. capsulatus* sensor strain S3. The mating process resulted in ~8 × 10^5^ clones per library, a number that required high-throughput screening methods.

### High-throughput selection of H_2_-overproducing *nifH* variants

**Figure 2:**
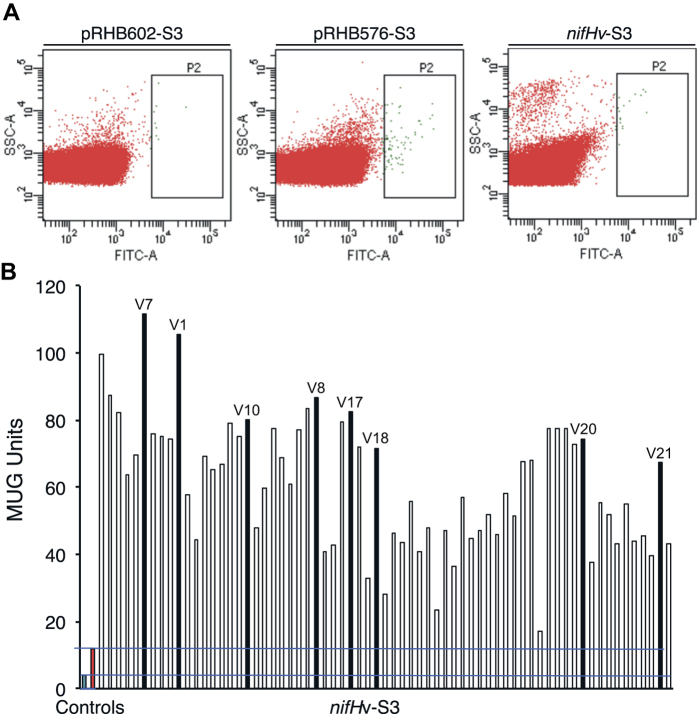
High-throughput selection of cells carrying H_2_-overproducing NifH variants. (**A**) Fluorescence-activated cell sorting. Dot-plot showing side-scattered light (SSC) versus fluorescence generated by fluorescein isothiocyanate (FITC) in pRHB602-S3 (no *nifH*), pRHB576-S3 (wild-type *nifH*), and *nifH*v-S3 (*nifH* variant pool) cell populations. P2 indicate the areas in which sorted cells exhibited fluorescence signals above the desired threshold. (**B**) β-Galactosidase activity of *nifH*v-S3 clones sorted by FACS as determined by MUG hydrolysis in a 96-well plate format. Blue and red bars represent pRHB602-S3 and pRHB576-S3 activities, respectively. Black bars represent activities of clones carrying *nifH* variants further selected to determine *in vivo* H_2_ production.

*R. capsulatus* S3 libraries expressing *nifH* variants (*nifH*v-S3) were grown under nitrogenase-derepressing conditions inside a glovebox. After treatment with fluorescein di-β-D-galactopyranoside (FDG), culture samples were screened by FACS flow cytometry. Approximately 2 × 10^5^ events were processed per sample. *R. capsulatus* cells emitting fluorescence at levels significantly higher than the main population (0.024% of total population, see P2 area in Fig. 2A) were sorted by the cytometer into separate wells of 96-well microplates containing growth medium.

S3 derivatives transformed with empty expression vector (pRHB602-S3) or wild-type *nifH*-containing expression vector (pRHB576-S3) were used as negative and positive controls, respectively. pRHB602-S3 populations emitted the lowest average level of fluorescence, and only 0.008% of the population was found above the set threshold in the P2 area. On the other hand, the average fluorescence emission in the pRHB576-S3 populations was the highest, although only 0.0039% of the population was above the selective threshold.

The *nifH*v-S3 cells sorted by FACS were then cultured under nitrogenase-derepressing conditions using serine as a poor nitrogen source inside a glovebox for a secondary MUG-based fluorescence screen. Growth was observed in approximately 25% of the inoculated wells. Figure 2B shows the β-galactosidase activity levels of 67 *nifH*v-S3 cultures along with those of the pRHB602-S3 (blue) and pRHB576-S3 (red) cultures used as controls. The β-galactosidase activity was 2 to 12-fold higher in the *nifH*v-S3 cultures than in the pRHB576-S3 strain (carrying wild-type *nifH*). Importantly, 85% of the *nifH*v-S3 clones harbored an expression vector with a *nifHv* gene, as determined by PCR analysis. The strong correlation of the results from the FACS primary screening and the secondary MUG screening in the 96-well format validates the use of flow cytometry as a high-throughput method to screen *nifH*v-S3 libraries.

### Nitrogenase-dependent H_2_ overproduction in selected strains with *nifH* variants

**Figure 3:**
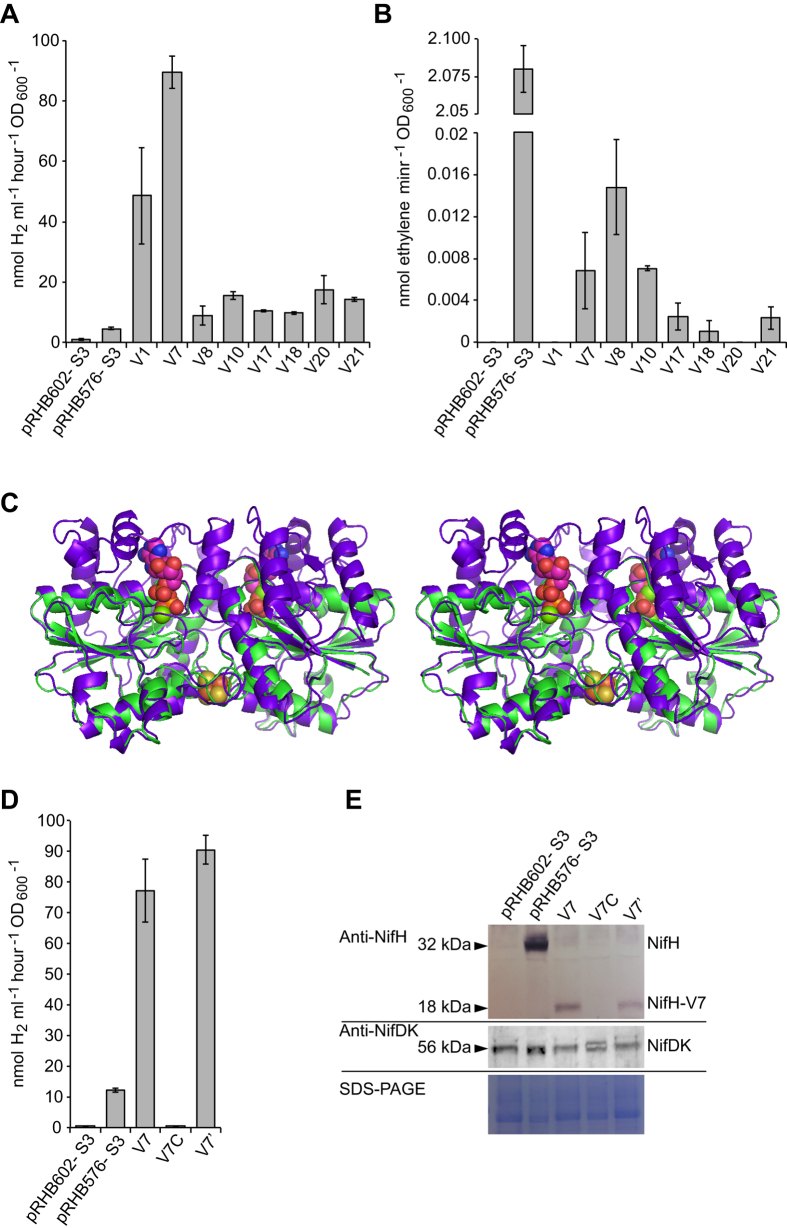
Characterization of selected *nifHv*-S3 strains. H_2_ production (**A**) and acetylene reduction activity (**B**) of highly fluorescent *R. capsulatus* strains carrying *nifH* variants. Strains pRHB602-S3 (no *nifH*) and pRHB576-S3 (wild-type *nifH*) were used as controls. Data represent the mean ± SD (n = 4). (**C**) Stereoview overlap of 3D-structural models for *R. capsulatus* NifH (purple) and the NifH-V7 variant (green). Both proteins are expected to form homodimers with a [4Fe-4S] cluster at the subunit interface. (**D**) Correlation of H_2_ production (nmol ml^−1^ hour^−1^) and presence of NifH-V7. Data represent the mean ± SD (n = 4). (**E**) Immunodetection of nitrogenase components NifH and NifDK in strains carrying or lacking the NifH-V7 variant. pRHB602-S3 and pRHB576-S3 were used as controls. Lower panel shows protein loading in each sample as determined by the Coomassie staining of the corresponding SDS gels.

Based on the β-galactosidase activity levels, *R. capsulatus* strains V1, V7, V8, V10, V17, V18, V20 and V21 (Fig. 2B, black bars), carrying the corresponding *nifH* variants, were selected for *in vivo* H_2_ production assays. All these strains produced more H_2_ than the pRHB576-S3 strain carrying wild-type *nifH* (Fig. 3A), with the V1 and V7 strains consistently producing up to 10-fold more H_2_. Importantly, the acetylene reduction activity was almost abolished in the strains carrying H_2_-overproducing *nifH* variants (Fig. 3B), a result consistent with the random mutagenesis process designed to select only for improved H_2_ production. The ratio of H_2_ to ethylene production in V7 strain was 6000-fold higher than in pRHB576-S3. Neither H_2_ nor ethylene was produced in the absence of *nifH* (see strain pRHB602-S3 in Fig. 3), indicating that the H_2_ production was dependent on the *nifH* expression.

The nature of the mutations in the *nifH*-V1 and *nifH*-V7 variants was determined by DNA sequencing (Table S2 and Fig. S7). A frame shift mutation was found in codon 172 of *nifH*-V7 that would result in a NifH protein lacking 124 amino acids at its C-terminus. In addition, NifH-V7 would carry G32C and A107T amino acid substitutions. This was surprising because NifH is a highly conserved protein, and such a drastic truncation would be expected to yield an inactive protein. An overlap of the three-dimensional models of *R. capsulatus* NifH and NifH-V7 is shown in Fig. 3C. The overall protein overlap (4–171 amino acid residues) and an RMSD value of 0.900 Angstroms for the protein backbone atoms indicate very similar model architectures. However, subtle differences were observed in the loops near the [4Fe-4S] cluster, where residues K43, A44, A100, G101, R102 and G115 did not overlap.

To confirm that the H_2_ overproduction in V7 was dependent on the activity of both Mo-nitrogenase component proteins, the following two experiments were performed. First, the *nifH*-V7 expression plasmid was cured from the V7 strain by passing five times under a nonselective media. Plasmid elimination was confirmed by PCR analysis and by growth inhibition on media supplemented with tetracycline (Tc). The resulting strain, V7C, was unable to produce H_2_ above the levels of the pRHB602-S3 control strain (0.42 nmol H_2_ h^−1^ OD_600_^−1^ ml^−1^) (Fig. 3D). The reintroduction of the *nifH*-V7 expression plasmid by mating completely restored the H_2_-producing activity (compare H_2_ productions of V7 and V7′ in Fig. 3D). The presence of a truncated form of NifH in V7 and its elimination in V7C were confirmed by an immunoblot analysis (Fig. 3E). This analysis also showed that all strains accumulated similar levels of nitrogenase component proteins, ruling out the possibility of increased accumulation underlying the H_2_ overproduction by V7.

Second, *R. capsulatus* sensor strains lacking either *nifH* (strain S3) or *nifHDK* (strain S5) were complemented with an expression plasmid harboring *nifH*-V7. While the S3-V7 cultures produced large amounts of H_2_, background levels were detected in the S5-V7 cultures (Fig. S8). This result demonstrates that the NifH-V7 dependent H_2_ production also requires the presence of NifDK and is not due to the activity of other cellular proteins, such as the Fe-only nitrogenase.

## Discussion

There are many reports of metabolic engineering aiming to increase microbial H_2_ production. Genetic approaches include deleting hydrogenase and/or nitrogenase structural and regulatory genes^20^,^21^,^22^,^23^,^24^,^25^, eliminating Rubisco^26^, lowering intracellular O_2_ levels^27^, re-engineering hydrogenase to increase its tolerance to O_2_^28^, and modifying nitrogenase substrate selectivity by site-directed mutagenesis^29^,^30^. Nonetheless, it has been difficult to find or engineer vastly improved H_2_-overproducing enzymes or microbial strains.

Directed evolution mimics biological evolution in the laboratory and is an effective approach to change enzyme properties such as catalytic turnover^31^. Directed evolution requires a strategy to generate libraries with a large number of variants and a method capable of screening or selecting for the best variants in a large pool. In this work, we have combined the random mutagenesis of *nifH* and high-throughput screening to improve nitrogenase H_2_-producing activity. We hypothesized that the nitrogenase H_2_ production could be greatly improved if no selection was applied to maintain the N_2_-reducing activity. The *nifH* gene was selected to obtain a proof of concept because the limiting steps in nitrogenase catalysis are the NifH/NifDK complex dissociation and the release of Pi from NifH^2^. FACS flow cytometry was used as a high-throughput method to select H_2_-overproducing NifH variants from the pool. A genetic module endowing a NifH-expressing sensor strain with the capacity to emit light in proportion to the amount of H_2_ detected was required for this selection. The screening of bacterial libraries by FACS flow cytometry had been performed before in directed evolution procedures^32^.

A number of H_2_-overproducing NifH variants were obtained. Perhaps the most interesting was the V7 variant, which lacks 124 amino acid residues at the C-terminus of the protein (Fig. 3C). In wild-type NifH, ATP binding and hydrolysis is required for electron transfer to NifDK during catalysis^2^. Interestingly, NifH-V7 lacks all residues shown to enable hydrogen bonding to the adenine base (Asp185, Gln218, and Gln236 in the *Azotobacter vinelandii* NifH), suggesting that NifH-V7 might have lost its specificity for Mg·ATP over other nucleotides^33^. The mechanism by which NifH-V7 is capable of sustaining H^+^ reduction merits further investigation.

Other biological and chemical methods have also been developed to detect H_2_-producing microorganisms. Chemochromic transparent sensor films that turn blue in the presence of H_2_ and indicators consisting of a coloring agent and a water-soluble derivative of Wilkinson’s catalyst have been used for phenotypic screenings of *Chlamydomonas reinhardtii*^34^,^35^ and *R. capsulatus*^36^, respectively. Moreover, a biosensor to screen algal H_2_ production was developed starting from the *R. capsulatus* H_2_-sensing system^37^,^38^. However, all these methods suffer from limitations in the number of clones that could be screened per experiment, making directed evolution techniques impossible to apply.

## Conclusions

In this work, we have re-engineered the natural H_2_ sensing system of *R. capsulatus* and combined it with FACS for the high-throughput selection of nitrogenase variants with enhanced H_2_ production that independently retain their N_2_ fixation activity. This method allows screening 10^5^–10^6^ variants per experiment, thus permitting enzyme improvement by directed evolution. This technology possesses great potential to identify nitrogenase amino acid substitutions leading to H_2_-overproducing variants that could be mimicked in nitrogenases from other microorganisms, expanding the impact of the findings. In addition, it might be used for the genome-wide screening of mutations leading to enhanced H_2_ production in *R. capsulatu*s.

## Materials and Methods

### β-galactosidase activity assays

Transcription from P*hupA* was estimated by measuring β-galactosidase activity of *R. capsulatus* strains carrying P*hupA*::*lacZ* transcriptional fusions. Cells were cultured at 30 °C for 7 h, transferred to assay tubes (0.7 ml of culture), permeabilized by addition of 20 μl of chloroform and 10 μl of 0.1% SDS, and β-galactosidase activity estimated at 28 °C as described^39^.

When the 96-well microplate format was used, β-galactosidase activity assays were carried out as described in^19^ with modifications. *R. capsulatus* cultures were incubated overnight under diazotrophic conditions inside a glove box in a 96-well plate (black/clear Optilux™ flat bottom; BD Biosciences) covered with a transparent adhesive sealer. One hundred and twenty μl of each culture were transferred to a 96-well microplate containing 100 μl of Z-Buffer in each well^39^, then supplemented with 25 μl of 4-methylumbelliferone β-D-galacto-pyranoside (MUG; 1 mg/ml stock solution in dimethyl sulfoxide), and incubated at room temperature for 2 h in darkness. MUG hydrolysis by β-galactosidase was quantified by fluorescence emission at 445 nm (372 nm excitation wavelength) in a Genios Pro (Tecan) microplate fluorometer.

### Construction of random mutagenesis libraries

Random mutagenesis of *nifH* was carried out by Error-Prone PCR using PCR GeneMorph^®^ II Random Mutagenesis Kit (Stratagene) according to the manufacturer’s instructions. Reaction mixtures contained 1 μl DNA template (0.4 ng of pRHB529 including 0.1 ng of *nifH*), 5 μl of 10X Mutazyme II reaction buffer, 1 μl of 40 mM dNTP mix (200 μM each final), 0.5 μl of primer mix (250 ng/μl of each primer), 1 μl of Mutazyme II DNA polymerase (2.5 U/μl), and 41.5 μl of H_2_O. Primers P19 and P20 were used to amplify *nifH* gene by Mutazyme II DNA polymerase. PCR conditions used were: 95 °C for 2 min, followed by 30 cycles of 95 °C for 30 sec, 55 °C for 30 sec, and 72 °C for 1 min, and finished with an incubation at 72 °C for 10 min. Amplified DNA was digested with *Nde*I and *Xba*I, ligated into pRHB602, and introduced into *E. coli* DH5α competent cells (NEB, C2987I) by heat shock to generate a expression library of *nifH* variants. On average, ~ 4 × 10^6^ transformants were recovered per 100 ng DNA.

### *In vivo* hydrogenase activity assays

Hydrogenase activity was measured by using a Clark-type hydrogen microelectrode (Unisense) with O_2_ as electron acceptor^40^. When necessary, hydrogenase expression was induced by injecting 9 ml of H_2_ into 100 ml-capped vials containing 10 ml of *R. capsulatus* cultures and incubating under culture conditions for 6 h.

### *In vivo* nitrogenase activity assays

To determine acetylene reduction activity in *R. capsulatus* cultures grown under diazotrophic conditions, 1-ml samples were transferred to 9-ml sealed vials with a 94% N_2_/6% acetylene gas phase and incubated at 30 °C in the light for 1 h. Ethylene formation was detected in 50 μl samples withdrawn from the gas phase by using a Shimadzu GC-2014 gas chromatographer equipped with a 9-ft long, 1/8-in diameter Porapak R column. *In vivo* nitrogenase activity units are defined as nmol ethylene formed per min per ml of culture at an OD_600_ equal to 1.

To determine H_2_ production in *R. capsulatus* cultures grown under diazotrophic conditions, 16-ml samples were transferred to 23-ml sealed vials with a 100% N_2_ atmosphere and incubated at 30 °C in the light for 48 h. H_2_ formation was detected in 250 μl samples withdrawn from the gas phase by using a Shimadzu GC-8A gas chromatographer equipped with a 6-ft long, 1/8-in diameter Molecular Sieve column. Activity units are defined as nmol H_2_ formed per h in a culture at an OD_600_ equal to 1.

### Protein methods

Protein concentration was determined by the bicinchoninic acid method (Pierce) with bovine serum albumin as the standard^41^. For SDS-PAGE, cells from 1-ml culture samples were collected by centrifugation, resuspended in 2 × Laemmli sample buffer supplemented with 0.1 M dithiothreitol (to a concentration equivalent to an OD_600_ of 4), and electrophoresed in 12% acrylamide/bisacrylamide (29:1) gels. For immunoblot analysis proteins were transferred to nitrocellulose membranes for 40 min at 20 V using a Transfer-Blot^®^ Semi Dry system (Bio-Rad). Immunoblot analyses were carried out with antibodies raised against a 1:1 mixture of *A. vinelandii* and *Rhodospirillum rubrum* NifH proteins (1:2,500 dilution) or with antibodies raised against *R. capsulatus* NifDK (1:2,000 dilution; antibody kindly donated by Yves Jouanneau, CNRS, Grenoble). Secondary alkaline phosphatase-conjugated anti-rabbit antibodies (Sigma, A3687) were used at 1:5,000 dilution.

### Flow cytometry

Cells from 50-ml *R. capsulatus* cultures under diazotrophic conditions inside a glove box were collected by centrifugation in Falcon tubes for 15 min at 4 °C, 4500 × *g*, resuspended in 5 ml PBS supplemented with 10% glycerol, and incubated for 30 min at 4 °C. Cells were then collected, resuspended in 1 ml of an 8:1:1 mixture of PBS, fluorescein di-β-D-galactopyranoside (FDG) and propidium iodide (PI), and incubated at 37 °C for 30 min to facilitate FDG entrance into the cells. FDG releases fluorescein when cleavage by β-galactosidase^42^. Cells were collected by centrifugation, resuspended in RCV medium supplemented with Tc, and analyzed in a FACSVantage (sorter) flow cytometer using an argon ion laser to excite the fluorochrome (488 nm). Cells exhibiting high fluorescence levels were sorted and recovered in 96-well microplates containing YPS medium supplemented with Tc.

### NifH 3-D models

3-D model of *R. capsulatus* NifH and the V7 variant were generated by homology modeling (http://swissmodel.expasy.org/) using the *A. vinelandii* NifH structures as template (1NIP for wild-type NifH and 1G5P for NifH-V7, respectively). Both models yielded NifH homodimer with capability to ligate [4Fe-4S] clusters.

## Additional Information

**How to cite this article**: Barahona, E. *et al*. Hydrogen overproducing nitrogenases obtained by random mutagenesis and high-throughput screening. *Sci. Rep.* **6**, 38291; doi: 10.1038/srep38291 (2016).

**Publisher’s note:** Springer Nature remains neutral with regard to jurisdictional claims in published maps and institutional affiliations.

## Supplementary Material

Supplementary Information
