# Corrigendum: Hydrogen overproducing nitrogenases obtained by random mutagenesis and high-throughput screening

**DOI:** 10.1038/srep43406

**Published:** 2017-02-24

**Authors:** Emma Barahona, Emilio Jiménez-Vicente, Luis M. Rubio

Scientific Reports 6: Article number: 3829110.1038/srep38291; published online: 12 02 2016; updated: 02 24 2017

This Article contains a typographical error in the Conclusions section.

“In this work, we have re-engineered the natural H_2_ sensing system of *R. capsulatus* and combined it with FACS for the high-throughput selection of nitrogenase variants with enhanced H_2_ production that independently retain their N_2_ fixation activity”.

should read:

“In this work, we have re-engineered the natural H_2_ sensing system of *R. capsulatus* and combined it with FACS for the high-throughput selection of nitrogenase variants with enhanced H_2_ production that might independently retain their N_2_ fixation activity”.

