# Supplementary material for: Hydrogen overproducing nitrogenases obtained by random mutagenesis and high-throughput screening
Source: Sci Rep. 2016 Dec 2;6:38291. doi: 10.1038/srep38291 (PMC5133592; doi:10.1038/srep38291)
Supplement: Supplementary Information [file srep38291-s1.pdf]

Article for *Scientific Reports*

(Revision submitted on 12-October-2016)

Supplementary Information to:

Hydrogen overproducing nitrogenases obtained by random mutagenesis and high-throughput screening

Emma Barahona, Emilio Jiménez-Vicente and Luis M. Rubio

Supplemental Materials and Methods

Bacterial strains and plasmids. Bacterial strains and plasmids used in this study are listed in Table S3.

Growth conditions. *R. capsulatus* strains were cultivated in either rich YPS [1] or minimal RCV [1] media under phototrophic (anaerobic) or chemotrophic (aerobic) conditions at ~30°C. Standard RCV medium contained 30 mM DL-malate and 10 mM $(NH_4)_2SO_4$ as sole carbon and nitrogen source, respectively. To derepress nitrogenase cells were either grown on RCV medium without combined nitrogen or supplemented with 9 mM serine, which serves as poor nitrogen source. Cells were cultured phototrophically inside a glove box with N_2 (< 500 ppm O_2). Media were supplemented with tetracycline (Tc) (0.8 μg/ml), kanamycin (Km) (50 μg/ml), spectinomycin (Sm) (10 μg/ml), gentamicin (Gm) (2.8 μg/ml) and rifampicin (Rif) (25 μg/ml) when required.

R. capsulatus cultures growing with exogenous H_2 were set up either in shake flasks (200 rpm) inside a glove box (Coy Labs, Michigan, USA) with 90% N_2 / 10% H_2, or by injecting 10% H_2 in the N_2 headspace of capped culture vials. Addition of exogenous H_2 was used to measure β-galactosidase activity and H_2 consumption.

Escherichia coli strains DH5α and S17.1 λpir were cultivated in Luria-Bertani medium at ~37ºC with shaking (200 rpm) and were used in routine cloning manipulations. Selective antibiotics used for *E. coli* were Tc (10 μg/ml), Km (50 μg/ml), Sm (25 μg/ml), Gm (10 μg/ml) and Ap (100 μg/ml).

Plasmid constructions and DNA manipulations. DNA purification, restriction enzyme digestion, plasmid constructions, PCR, *E. coli* transformation and other DNA techniques were carried out by standard methods [2]. Plasmids used in this study are listed in Table S3, and primers used for PCR amplifications are listed in Table S4.

The plasmid to introduce an in-frame *nifH* deletion into the *R. capsulatus* chromosome was generated as follows. Flanking regions of *nifH* gene were amplified by PCR using the oligonucleotides P1 and P2 for the region upstream of *nifH,* and P3 and P4 for the region downstream of *nifH*. The resulting PCR products were digested with *BamH*I and *EcoR*I and cloned into the *BamH*I site of pK18*mob* suicide vector [3] by quadruple-ligations together with an *EcoR*I-digested Gm-resistance cassette to generate pRHB541.

The plasmid to introduce an in-frame *nifHDK* deletion into the *R. capsulatus* chromosome was generated as follows. Upstream region of *nifH* gene was amplified by PCR using the oligonucleotides P1 and P2 and downstream region of *nifK* gene was also amplified using P21 and P22. PCR products were digested with *BamH*I and *EcoR*I and cloned into the *BamH*I site of pK18*mob* suicide vector by quadruple-ligations together with an *EcoR*I-digested Gm-resistance cassette to generate pRHB704.

The plasmid to introduce an in-frame *hupAB* deletion into the *R. capsulatus* chromosome was generated as follows. Flanking regions of *hupAB* genes were amplified by PCR using the oligonucleotides P5 and P6 for the region upstream and P7 and P8 for the region downstream. The resulting PCR products were digested with *BamH*I and *Hind*III and cloned into the *BamH*I site of a modified version of pK18*mobsacB*

(pSm18*mobsacB*) suicide vector previously digested with *BamH*I [3] by triple-ligation to generate pRHB577.

To introduce an in-frame *hupT* deletion from *R. capsulatus* chromosome, *hupT* flanking regions were amplified by PCR using oligonucleotides P9 and P10 for the region upstream and P11 and P12 for the region downstream. The resulting PCR products were digested with *Xba*I and *BamH*I and cloned into the *Xba*I site of pK18*mobsacB* plasmid by quadruple-ligations together with a *BamH*I-digested Gm-resistance cassette to generate plasmid pRHB627.

To introduce an in-frame *hupR* deletion from *R. capsulatus* chromosome, regions flanking *hupR* were PCR-amplified using oligonucleotides P13 and P14 for the region upstream and P15 and P16 for the region downstream. The resulting PCR products were digested with *Sal*I and *Hind*III and cloned into *Sal*I and *Hind*III sites of pK18*mobsacB* to generate plasmid pRHB552.

To construct the P*hupA*::*lacZ* transcriptional fusion, an 874-bp DNA fragment containing the *hupA* promoter (P*hupA*) [4] was amplified using the oligonucleotides P17 and P18 and cloned into the *Kpn*I and *Xba*I sites of replicative plasmid pMP220, yielding pRHB502. In addition, the digested PCR product was cloned into the *Kpn*I and *Xba*I sites of pVIK112 suicide vector [5] to generate pRHB501, which carries a chromosomal translational fusion.

A 681-bp DNA fragment containing *R. capsulatus nifH* promoter (P*nifH*) followed by *Nde*I and *Xba*I restriction sites [6] and flanked by transcriptional terminators was synthesized (GenScript, USA) and cloned into the *Kpn*I and *Sac*I sites of the broad-host-range cloning vector pBBR1MCS-3 [7] to generate pRHB602. *R. capsulatus nifH* gene

was amplified using primers P19 and P20, digested using *Nde*I and *Xba*I restriction enzymes and cloned into pRHB602 to generate pRHB576. The same PCR product comprising the amplification of a full *nifH* gene was cloned into the *Nde*I and *Xba*I sites of pUC18 to generate pRHB529, used as a template in Error-Prone assays. The *nifH, nifD* and *nifK* were amplified together from *R. capsulatus* genomic DNA using the oligonucleotides P19 and P23 digested using *Nde*I and *Xba*I restriction enzymes and cloned into pRHB602 to generate pRHB618. All DNA constructions were confirmed by restriction analysis and DNA sequencing.

Generation and analysis of *R. capsulatus* mutant strains. Plasmids were introduced into *R. capsulatus* strains by biparental or triparental mating. Biparental matings were performed by culturing a mixture of *E. coli* S17.1 λpir donor cells harboring the desired plasmid and *R. capsulatus* as recipient cells at a 1:2 ratio. Mixtures were cultured overnight at 30ºC over nitrocellulose filters atop YPS-containing plates. Triparental matings were performed by culturing 1:1:2 mixtures of an *E. coli* helper strain (carrying either helper plasmid pRK2013 [8] or pRK600 [9]), a donor *E. coli* DH5α strain carrying a plasmid to be transferred, and *R. capsulatus* serving as recipient cells. Mixtures were cultured overnight at 30ºC over nitrocellulose filters atop YPS-containing plates. Transconjugant *R. capsulatus* colonies were selected on YPS plates supplemented with the corresponding selective antibiotics. Plasmid transfer and incorporation of mutations into the chromosome of recipient *R. capsulatus* cells were screened by PCR.

When pK18*mobsacB* or pSm18mobsacB were used to generate mutants, putative single recombinants were selected by their resistance to Sm or Km, and then isolated

from the agar surface and inoculated in YP liquid medium. Cells were passaged five times under no selective pressure to undergo a second recombination event. After passaging, different dilutions (10^{-1}, 10^{-2}, 10^{-3}, and 10^{-4}) of the final culture were spread on YPS plates containing 10% (w/v) of sucrose. Double recombinant colonies were selected due to their capacity to grow in presence of 10% of sucrose.

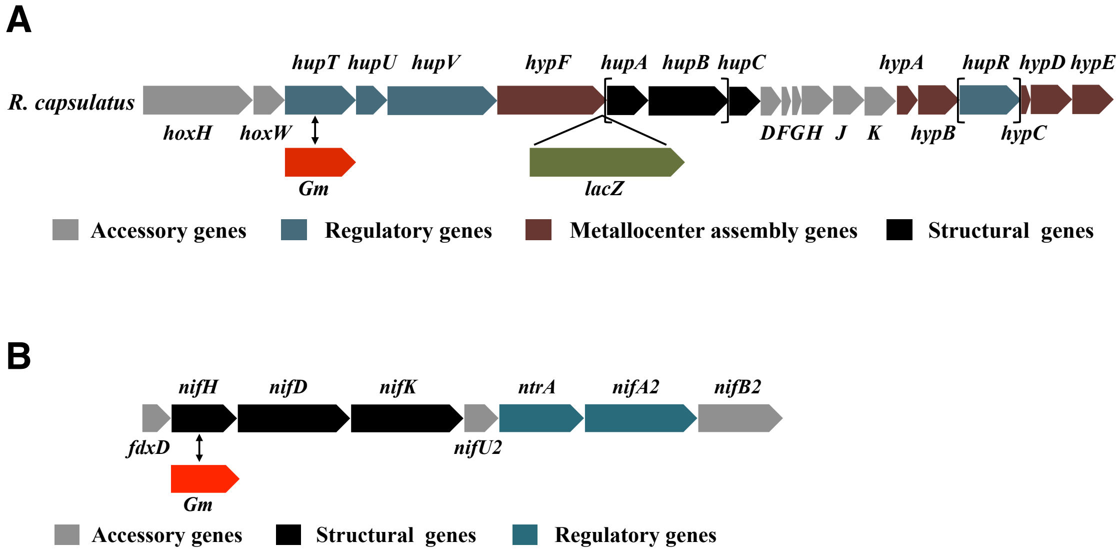

Fig. S1. *R. capsulatus hup-hyp* (*A*) and *nif* (*B*) gene cluster structures. Location of the *lacZ* reporter cassette and mutation sites leading to the *hupAB*, *hupT*, *hupR* , and *nifH* mutants are indicated by brackets (deletions) or by arrows (insertion of antibiotic resistance cassette).

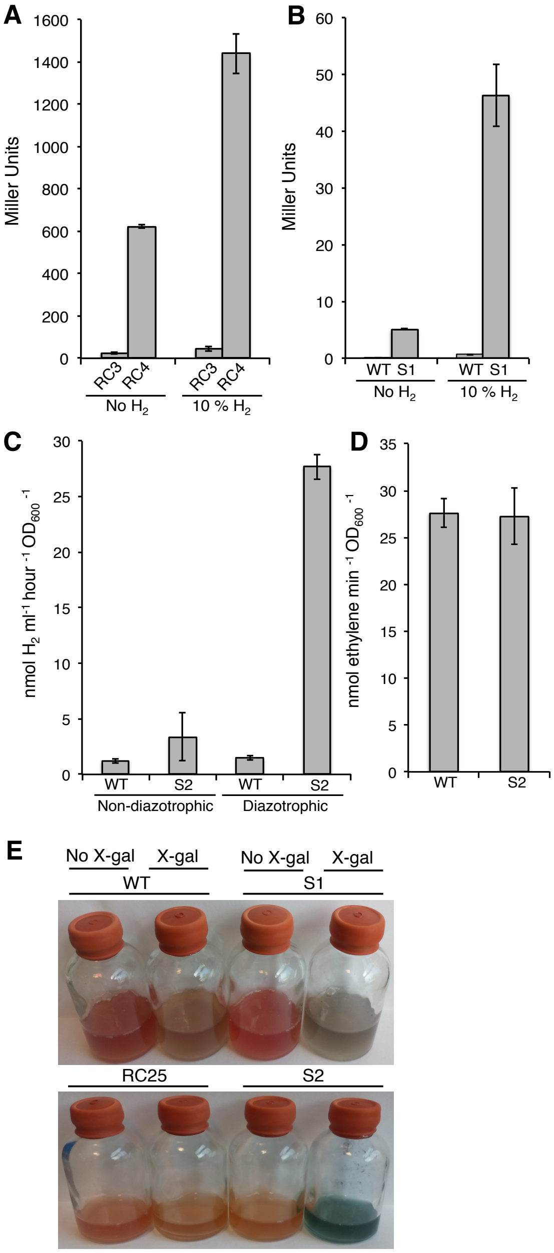

Fig. S2. Characterization of *R. capsulatus* H_2 reporter strains. β-galactosidase activity determinations in the absence or presence of 10% H_2 in the culture gas phase (*A* and *B*). Strain RC3 carries reporter plasmid pMP220; strain RC4 carries plasmid pRHB502 (P*hupA::lacZ* in pMP220). Strain S1 carries the P*hupA::lacZ* fusion integrated into the chromosome. (*C*) *In vivo* nitrogenase activity of WT and S2 measured as levels of ethylene production (nmol min^{-1}). (*D*) H2 production (nmol ml^{-1} hour^{-1}) by WT and S2 in non-diazotrophic and diazotrophic conditions. (*E*) Colorimetric assays using X-gal to measure the response to H_2 in WT, S1, RC25 and S2 cultures growing in the presence of 10% of H_2. WT and RC25 parental strains are used as controls.

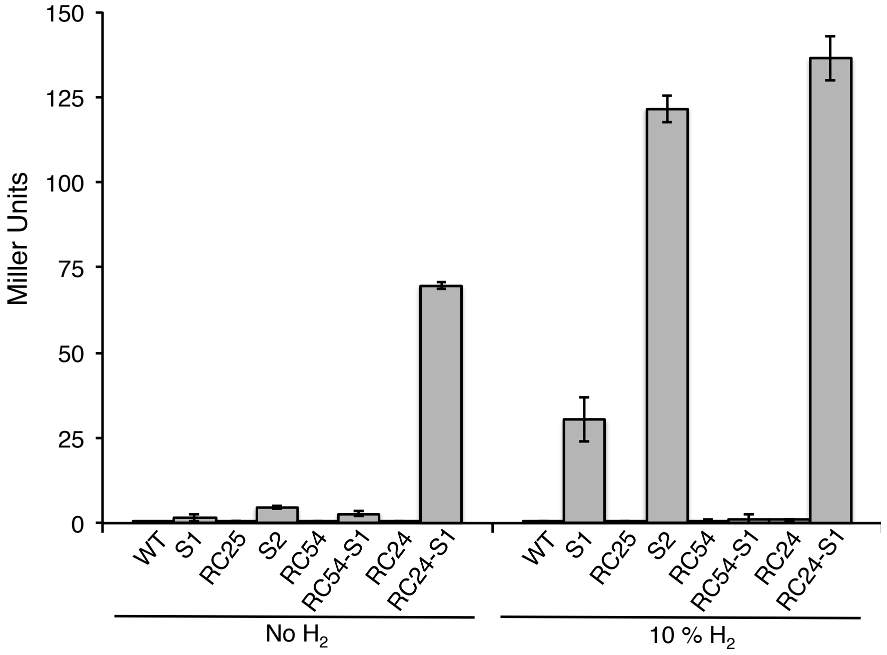

Fig. S3. β-Galactosidase activity determination in *R. capsulatus* reporter strains with mutations in H_2 signal transduction and metabolism pathways. β-galactosidase activity was determined either in the absence or presence of 10% of H_2 in the gas phase. Strain genotypes are described in Supplementary Table 2.

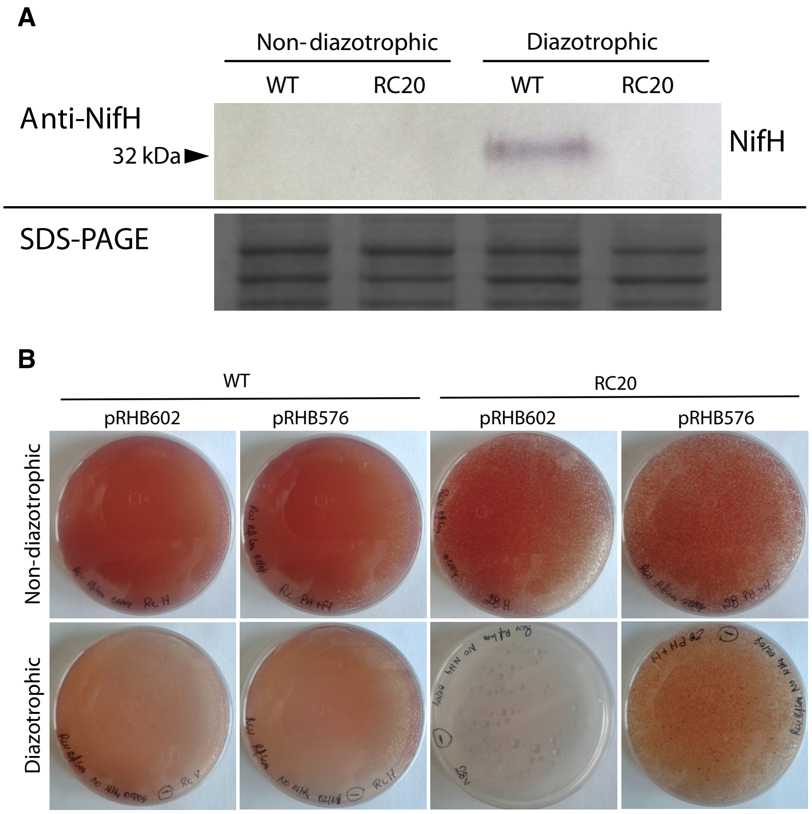

Fig. S4. NifH expression and diazotrophic growth analysis of RC20 strain. (*A*) NifH Immunodetection in WT and RC20 (Δ*nifH*) strains grown under non-diazotrophic or diazotrophic conditions. Lower panel shows Comassie-blue general staining of proteins as protein loading control. RC20 was used to generate S3 reporter strain (S3 genealogy is WT→RC20→RC31→S3). (*B*) RC20 genetic complementation assay. Plasmids pRHB602 (P*nifH*) and pRHB576 (P*nifH*::*nifH*) were introduced into WT and RC20 strains. Transformed cultures were then plated on RCV media supplemented with ammonium (non-diazotrophic) or lacking ammonium (diazotrophic).

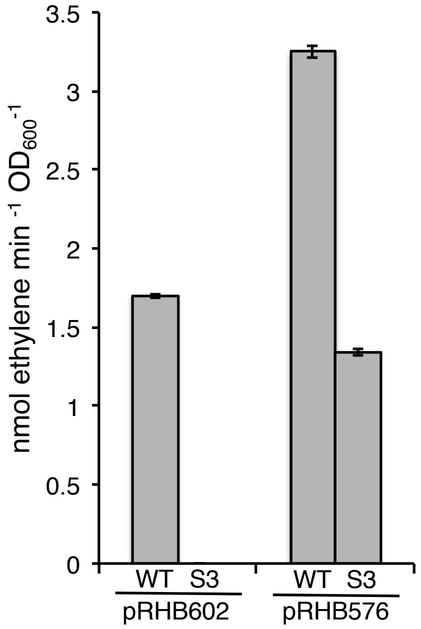

Fig. S5. Elimination of nitrogenase activity in S3. Determination of *in vivo* acetylene reduction activity of S3 strain carrying expression vectors pRHB602 (P*nifH*) or pRHB576 (*PnifH*::*nifH*). Cultures of the wild-type strain carrying either pRHB602 or pRHB576 were used as control.

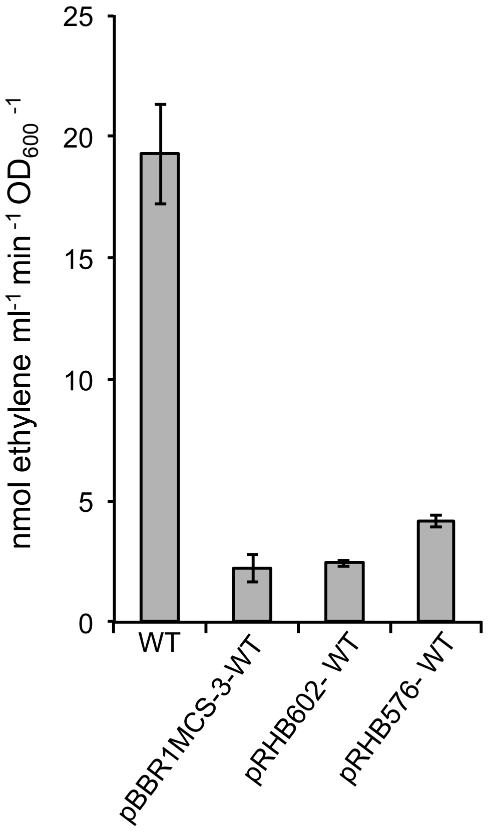

Fig. S6. Effect of expression vector pBBR1MCS-3 and its derivatives on *R. capsulatus in vivo* nitrogenase activity. In vivo acetylene reduction activities of WT and derivative strains carrying expression vectors pBBR1MCS-3, pRHB602 (P*nifH*) or pRHB576 (P*nifH*::*nifH*).

```
nifH wt    ATGGGCAAACTCCGTCAGATCGCCTTCTACGGCAAAGGTGGTATCGGCAAGTCGACCACC
nifH-V7    ATGGGCAAACTCCGTCAGATCGCCTTCTACGGCAAAGGTGGTATCGGCAAGTCGACCACC
           ************************************************************
nifH wt    TCGCAGAACACCCTCGCCGCGCTGGTCGAGATGGGTCAGAAGATCCTCATCGTCGGCTGC
nifH-V7    TCGCAGAACACCCTCGCCGCGCTGGTCGAGATGTGTCAGAAGATCCTCATCGTCGGCTGC
           ********************************* **************************
nifH wt    GACCCCAAGGCTGACAGCACCCGTCTGATCCTGAACACCAAGCTGCAGGACACCGTGCTG
nifH-V7    GACCCCAAGGCTGACAGCACCCGTCTGATCCTGAACACCAAGCTGCAGGACACCGTGCTG
           ************************************************************
nifH wt    CACCTGGCCGCCGAGGCCGGTTCGGTCGAAGATCTGGAAGTCGAAGACGTCGTGAAAATC
nifH-V7    CACCTGGCCGCCGAGGCCGGTTCGGTCGAAGATCTGGAAGTCGAAGACGTCGTGAAAATC
           ************************************************************
nifH wt    GGCTACAAGGGCATCAAATGCACCGAAGCCGGCGGTCCGGAGCCGGGGGTTGGCTGCGCC
nifH-V7    GGCTACAAGGGCATCAAATGCACCGAAGCCGGCGGTCCGGAGCCGGGGGTTGGCTGCGCC
           ************************************************************
nifH wt    GGCCGTGGCGTCATCACCGCGATCAACTTCCTTGAAGAAAACGGCGCCTATGACGATGTG
nifH-V7    GGCCGTGGCGTCATCACCACGATCAACTTCCTTGAAGAAAACGGCGCCTATGACGATGTG
           ****************** *****************************************
nifH wt    GACTATGTGTCCTATGACGTTCTGGGCGACGTGGTCTGCGGCGGCTTCGCCATGCCGATC
nifH-V7    GACTATGTGTCCTATGACGTTCTGGGCGACGTGGTCTGCGGCGGCTTCGCCATGCCGATC
           ************************************************************
nifH wt    CGTGAAAACAAGGCGCAGGAAATCTACATCGTCATGTCGGGCGAGATGATGGCGCTTTAC
nifH-V7    CGTGAAAACAAGGCGCAGGAAATCTACATCGTCATGTCGGGCGAGATGATGGCGCTTTAC
           ************************************************************
nifH wt    GCCGCCAACAACATCGCCAAGGGCATCCTGAAATATGCGAACTCGGGCGGCGTGCGTCTG
nifH-V7    GCCGCCAACAACATCGCCAAGGGCATCCTGAAATAAGCGAACTCGGGCGGCGTGCGTCTG
           *********************************** ************************
nifH wt    GGCGGGCTGATCTGCAACGAACGCAAGACCGACCGCGAGCTGGAACTGGCCGAAGCGCTG
nifH-V7    GGCGGGCTGATCTGCAACGAACGCAAGACCGACCGCGAGCTGGAACTGGCCGAAGCGCTG
           ************************************************************
nifH wt    GCCGCCAAGCTGGGCTGCAAGATGATCCACTTCGTGCCGCGCAACAACGTCGTGCAACAT
nifH-V7    GCTGCCAAGCTGGGCTGCAAGATGATCCACTTCGTGCCGCGCAACAACGTCGTGCAACAT
           ** *********************************************************
nifH wt    GCCGAACTGCGCCGCGAAACCGTGATCCAATACGATCCGACCTGCAGCCAGGCGCAGGAA
nifH-V7    GCCGAACTGCGCCGCGAAACCGTGATCCAATACGATCCGACCTGCAGCCAGGCGCAGGAA
           ************************************************************
nifH wt    TACCGCGAACTGGCCCGCAAGATCCACGAGAACTCGGGCAAGGGCGTCATCCCGACCCCG
nifH-V7    TACCGCGAACTGGCCCGCAAGATCCACGAGAACTCGGGCAAGGGCGTCATCCCGACCCCG
           ************************************************************
nifH wt    ATCACGATGGAAGAGCTGGAAGAGATGCTGATGGATTTCGGCATCATGCAATCGGAAGAA
nifH-V7    ATCACGATGGAAGAGCTGGAAGAGATGCTGATGGATTTCGGCATCATGCAATCGGAAGAA
           ************************************************************
nifH wt    GATCGCGAAAAGCAGATCGCCGAGATGGAAGCCGCGATGAAGGCCTGA
nifH-V7    GATCGCGAAAAGCAGATCGCCGAGATGGAAGCCGCGATGAAGGCCTGA
```

Fig. S7. Nucleotide sequence alignment between the *nifH* wt and *nifH*-V7 genes. Nucleotide identities are marked by *. Nucleotide changes are noted in red in the *nifH*-V7 sequence. The change that introduces a stop codon in the *nifH*-V7 sequence is underlined.

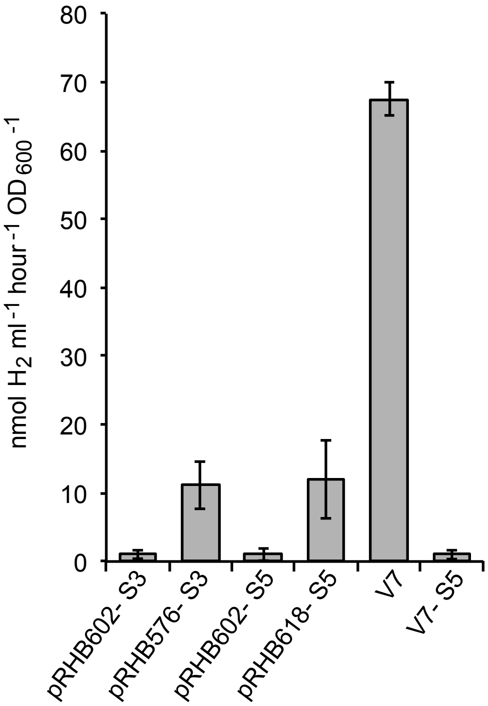

Fig. S8. NifDK is required for NifH-V7-dependent H_2 producion. In vivo H_2 production activities of S3 (*ΔnifH ΔhupAB* P*hupA::lacZ*) and S5 (*ΔnifHDK ΔhupAB* P*hupA::lacZ*) derivative strains carrying expression vectors pRHB602 (P*nifH*), pRHB576 (P*nifH*::*nifH*), pRHB618 (P*nifH*::*nifHDK*), or V7 (P*nifH*::*nifH-V7*).

Table S1. Results of *nifH* amplification by error-prone PCR

A. Mutation type and frequency

Transitions (Ts)	Transversions (Tv)	Mutation frequency (mutations/kb)	Ts/Tv
61.8%	38.2%	16.33	1.81

B. Average amino acid changes

Average amino acid changes per kb	Average amino acid changes in *nifH*
5.25	4.66

Table S2. Mutations in *nifH*-V1 and *nifH*-V7 variants

Variant	Nucleotide changes	Amino acid substitutions
nifH-V1	141C>T, 143G>C, 374A>G, 509T>A, 660C>T	R48P, I80F, L170Q
nifH-V7	94G>T, 319G>A, 516T>A, 603C>T	G32C, A107T, Y172*

*stop codon

Table S3. Strains and plasmids used in this work

R. capsulatus	Characteristics	Source/ Reference
SB1003 (WT)	Spontaneous Rif^R mutant derived from B10 wild-type strain	[10]
RC3	WT strain carriying the replicative plamid pMP220, Rif^R, Tc^R	This work
RC4	WT strain carrying the replicative plamid pRHB502, Rif^R, Tc^R	This work
RC25	Δ*hupAB*, mutant lacking *hupA* and *hupB* genes, Rif^R.	This work
RC54	Δ*hupR*, mutant lacking *hupR*, Rif^R	This work
RC24	Δ*hupT*, mutant lacking *hupT*, Rif^R	This work
S1	WT harboring a chromosomal translational fusion, P*hupA::lacZ*, Rif^R, Km^R	This work
S2	Δ*hupAB* harboring a chromosomal translation fusion, P*hupA::lacZ*, Rif^R, Km^R	This work
RC54-S1	Δ*hupR*, harboring a chromosomal translation fusion, P*hupA::lacZ*, Rif^R, Km^R	This work
RC24-S1	Δ*hupT*, harboring a chromosomal translation fusion, P*hupA::lacZ*, Rif^R, Km^R	This work
RC20	Δ*nifH*, mutant lacking *nifH* gene, Rif^R, Gm^R	This work
RC31	Δ*hupAB* Δ*nifH* double mutant, Rif^R, Gm^R	This work
RC43	Δ*hupAB* Δ*nifHDK* double mutant, Rif^R, Gm^R	This work
S3	Δ*hupAB* Δ*nifH* double mutant harboring a chromosomal translational fusion P*hupA::lacZ*, Rif^R, Gm^R, Km^R	This work
S5	Δ*hupAB* Δ*nifHDK* double mutant harboring a chromosomal translational fusion P*hupA::lacZ*, Rif^R, Gm^R, Km^R	This work
pBBR1MCS-3-WT	WT carrying pBBR1MCS-3, Rif^R,Tc^R	This work
pRHB602-WT	WT carrying pBBR1MCS-3 containing P*nifH* Rif^R, Tc^R	This work
pRHB576-WT	WT carrying pBBR1MCS-3 containing a *PnifH*::*nifH* fusion, Rif^R, Tc^R	This work
pRHB602-S3	S3 carrying pBBR1MCS-3 containing P*nifH* Rif^R, Tc^R, Gm^R, Km^R	This work
pRHB576-S3	S3 carrying pBBR1MCS-3 containing a P*nifH::nifH* fusion, Rif^R, Tc^R, Gm^R, Km^R	This work
pRHB602-S5	S5 carrying pBBR1MCS-3 containing P*nifH* Rif^R, Tc^R, Gm^R, Km^R	This work
pRHB618-S5	S5 carrying pBBR1MCS-3 containing a *PnifH*::*nifHDK* fusion, Rif^R, Tc^R, Gm^R, Km^R	This work
V1	S3 carrying pRHB602-V1, Rif^R, Tc^R, Gm^R, Km^R	This work
V7	S3 carrying pRHB602-V7, Rif^R, Tc^R, Gm^R, Km^R	This work
V8	S3 carrying pRHB602-V8, Rif^R, Tc^R, Gm^R, Km^R	This work
V10	S3 carrying pRHB602-V10, Rif^R, Tc^R, Gm^R, Km^R	This work
V18	S3 carrying pRHB602-V18, Rif^R, Tc^R, Gm^R, Km^R	This work
V20	S3 carrying pRHB602-V20, Rif^R, Tc^R, Gm^R, Km^R	This work
V21	S3 carrying pRHB602-V21, Rif^R, Tc^R, Gm^R, Km^R	This work
V7C	V7 cured of pRHB602-V7, Rif^R, Gm^R, Km^R	This work
V7'	V7C carrying pRHB602-V7, Rif^R, Tc^R, Gm^R, Km^R	This work
V7-S5	S5 carriying pRHB602-V7, Rif^R, Tc^R, Gm^R, Km^R	This work

E. coli	Characteristics	Source/ Reference
DH5α	Cloning strain	Gibco – BRL
S17-1 λpir	Suitable for biparental mating	[11]

Plasmid	Features	Reference
pK18*mob*	Suicide vector, mobilizable (*oriT*), Km^R	[3]
pK18*mobsacB*	Suicide vector, mobilizable (*oriT*), *sacB,* Km^R	[3]
pSm18*mobsacB*	Suicide vector, mobilizable (*oriT*), *sacB,* Sm^R	This work
pVIK112	Suicide plasmid containing promoter-less *lacZ*, Km^R	[5]
pMP220	Replicative plasmid containing promoterless *lacZ*, Tc^R	[12]
pBBR1MCS-3	Broad-host-range cloning vector	[7]
pRK600	Helper plasmid for matings	[9]
pRK2013	Helper plasmid for matings	[8]
pRHB541	To delete *nifH* gene. Derived from pK18*mobsacB*, Km^R	This work
pRHB577	To delete *hupAB* genes. Derived from pSm18*mobsacB*, Sm^R	This work
pRHB627	To delete *hupT* gene. Derived from pK18*mobsacB*, Km^R	This work
pRHB552	To delete *hupR* gene. Derived from pK18*mobsacB*, Km^R	This work
pRHB704	To delete *nifHDK*. Derived from pK18*mobsacB*, Km^R	This work
pRHB502	pMP220 containing a P*hupA::lacZ* fusion, Tc^R	This work
pRHB501	To introduce the P*hupA::lacZ* fusion into the chromosome of *R. capsulatus* strains. Derived from pVIK112, Km^R	This work
pRHB602	pBBR1MCS-3 containing P*nifH*, Tc^R	This work
pRHB576	pBBR1MCS-3 containing the P*nifH::nifH* fusion, Tc^R	This work
pRHB529	pUC18 containing *nifH* as template for error-prone PCR, Ap^R	This work
pRHB618	pBBR1MCS-3 containing the P*nifH::nifHDK* fusion, Tc^R	This work

Table S4. **Primers used in this work**

Primer	Sequence	RE site	Used for
P1	GGCGGATCCCACCGCCGAGGAGATCGA	*BamH*I	*nifH* deletion
P2	GCCGAATTCGCCCATGTGTGGCTCCCT	*EcoR*I	
P3	GCCGAATTCGGCAGCCTGCCCACCTTT	*EcoR*I	
P4	GGCGGATCCCGTGGCTGATGTGGACCAT	*BamH*I	
P5	GGCGGATCCGCCAAAGCTACGAGGGCG	*BamH*I	*hupAB* deletion
P6	CCGAAGCTTTTGTCCCTCCCTTGCCTTG	*Hind*III	
P7	CCGAAGCTTGGGGATGCCATGAAGGGA	*Hind*III	
P8	GGCGGATCCGCCGAACAGGCTGTCGG	*BamH*I	
P9	GCCTCTAGAGCTGCGTTCGATCCTAGG	*BamH*I	*hupT* deletion
P10	GCCGGATCCCTTGACTGGCAGTCGGGG	*Xba*I	
P11	GCCGGATCCGCATGAAGGTTCTGTGGTTG	*Xba*I	
P12	GCCTCTAGAGCGGGCAACCATGATGCA	*BamH*I	
P13	GGTGTCGACGGCCATTCCCATGGCGC	*Sal*I	*hupR* deletion
P14	GGCAAGCTTTCAGCGCGCTTTCCGCG	*Hind*III	
P15	CCGAAGCTTATGTGTCTGGGGATTCCGG	*Hind*III	
P16	CCGTCGACGCGTCGTCGTCTCGAAGCC	*Sal*I	
P17	GGTGGTACCGCTGCTGGGCGATTACAAG	*Kpn*I	P*hupA*
P18	GCCTCTAGACGACATTTGTCCCTCCCTT	*Xba*I	
P19	GGTCATATGATGGGCAAACTCCGTCAGAT	*Nde*I	*nifH gene*
P20	GCCTCTAGACAGGCCTTCATCGCGGC	*Xba*I	
P21	GCCGAATTCGCTTCGATCTGACCCGCT	*EcoR*I	*nifHDK deletion*
P22	GGCGGATCCGCGCATCTCGGCGATCAG	*BamH*I	
P23	GCCTCTAGATCAGCGGGTCAGATCGAAG	*Xba*I	

References

1 Wall, J. D., Weaver, P. F. & Gest, H. Gene transfer agents, bacteriophages, and bacteriocins of *Rhodopseudomonas capsulata*. *Arch. Microbiol.* **105**, 217-224, (1975).

2 Sambrook, J. & Russell, D. W. Molecular Cloning: A Laboratory Manual, Volume 1. 2344, (2001).

3 Schäfer, A. *et al.* Small mobilizable multi-purpose cloning vectors derived from the *Escherichia coli* plasmids pK18 and pK19: selection of defined deletions in the chromosome of *Corynebacterium glutamicum*. *Gene* **145**, 69-73, (1994).

4 Dischert, W., Vignais, P. M. & Colbeau, A. The synthesis of *Rhodobacter capsulatus* HupSL hydrogenase is regulated by the two-component HupT/HupR system. *Mol. Microbiol.* **34**, 995-1006, (1999).

5 Kalogeraki, V. S. & Winans, S. C. Suicide plasmids containing promoterless reporter genes can simultaneously disrupt and create fusions to target genes of diverse bacteria. *Gene* **188**, 69-75, (1997).

6 Pollock, D., Bauer, C. E. & Scolnik, P. A. Transcription of the *Rhodobacter capsulatus nifHDK* operon is modulated by the nitrogen source. Construction of plasmid expression vectors based on the *nifHDK* promoter. *Gene* **65**, 269-275, (1988).

7 Kovach, M. E. *et al.* Four new derivatives of the broad-host-range cloning vector pBBR1MCS, carrying different antibiotic-resistance cassettes. *Gene* **166**, 175-176, (1995).

8 Figurski, D. H. & Helinski, D. R. Replication of an origin-containing derivative of plasmid RK2 dependent on a plasmid function provided in trans. *Proc. Natl. Acad. Sci. U. S. A.* **76**, 1648-1652, (1979).

9 Finan, T. M., Kunkel, B., De Vos, G. F. & Signer, E. R. Second symbiotic megaplasmid in *Rhizobium meliloti* carrying exopolysaccharide and thiamine synthesis genes. *J. Bacteriol.* **167**, 66-72, (1986).

10 Cullen, P. J., Kaufman, C. K., Bowman, W. C. & Kranz, R. G. Characterization of the *Rhodobacter capsulatus* housekeeping RNA polymerase. In vitro transcription of photosynthesis and other genes. *J. Biol. Chem.* **272**, 27266-27273, (1997).

11 Simon, R., Priefer, U. & Puhler, A. A broad host range mobilization system for in vivo genetic engineering: transposon mutagenesis in gram-negative bacteria. *Nat. Biotechnol.* **1**, 784-791, (1983).

12 Spaink, H. P., Okker, R. J., Wijffelman, C. A., Pees, E. & Lugtenberg, B. J. Promoters in the nodulation region of the *Rhizobium leguminosarum* Sym plasmid pRL1JI. *Plant Mol. Biol.* **9**, 27-39, (1987).
